# Exploring the relationship between mobility and COVID− 19 infection rates for the second peak in the United States using phase-wise association

**DOI:** 10.1186/s12889-021-11657-0

**Published:** 2021-09-14

**Authors:** Raju Gottumukkala, Satya Katragadda, Ravi Teja Bhupatiraju, Azmyin Md. Kamal, Vijay Raghavan, Henry Chu, Ramesh Kolluru, Ziad Ashkar

**Affiliations:** 1 Informatics Research Institute, University of Louisiana at Lafayette, Lafayette, USA; 2 Mechanical Engineering Department, University of Louisiana at Lafayette, Lafayette, USA

## Abstract

Human mobility plays an important role in the dynamics of infectious disease spread. Evidence from the initial nationwide lockdowns for COVID− 19 indicates that restricting human mobility is an effective strategy to contain the spread. While a direct correlation was observed early on, it is not known how mobility impacted COVID− 19 infection growth rates once lockdowns are lifted, primarily due to modulation by other factors such as face masks, social distancing, and the non-linear patterns of both mobility and infection growth. This paper introduces a piece-wise approach to better explore the phase-wise association between state-level COVID− 19 incidence data and anonymized mobile phone data for various states in the United States. Prior literature analyzed the linear correlation between mobility and the number of cases during the early stages of the pandemic. However, it is important to capture the non-linear dynamics of case growth and mobility to be usable for both tracking and forecasting COVID− 19 infections, which is accomplished by the piece-wise approach. The associations between mobility and case growth rate varied widely for various phases of the epidemic curve when the stay-at-home orders were lifted. The mobility growth patterns had a strong positive association of 0.7 with the growth in the number of cases, with a lag of 5 to 7 weeks, for the fast-growth phase of the pandemic, for only 20 states that had a peak between July 1st and September 30, 2020. Overall though, mobility cannot be used to predict the rise in the number of cases after initial lockdowns have been lifted. Our analysis explores the gradual diminishing value of mobility associations in the later stage of the outbreak. Our analysis indicates that the relationship between mobility and the increase in the number of cases, once lockdowns have been lifted, is tenuous at best and there is no strong relationship between these signals. But we identify the remnants of the last associations in specific phases of the growth curve.

## Introduction

COVID− 19 has spread rapidly worldwide, nearing 99 million confirmed cases, and more than 2.1 million deaths were reported globally as of January 25, 2021 [1]. Public health officials continue to promote social distancing, face masks, and handwashing as effective mechanisms to contain the COVID− 19 outbreak [2], especially due to delays in mass vaccination and the growing number of new COVID− 19 strains [3]. The importance of tracking human mobility as an essential measure to understand and predict the spread of COVID− 19 has been highlighted by many prior studies [2, 4–7]. Local governments continue to track human mobility in their communities through anonymized cell phone data made available through various data providers [8–10]. Several prior studies [11–20] report a strong association between mobility and stay-at-home orders. Further analysis by Gatalo et al. [21] found that the early association between mobility and COVID− 19 incidence withered after the analysis was expanded to later epochs. This weakening association was likely due to non-pharmaceutical interventions such as face masks, handwashing, maintaining physical distance, avoiding large gatherings, and school closings.

The incidence rate of COVID− 19 disease does not follow a linear pattern, but rather follows a pattern of rise and fall, i.e., a logistic or power-law pattern, depending on the community’s response to contain the spread [22–25]. Similarly, as communities started to reopen, mobility also does not follow a linear pattern. It is important to capture the association of mobility and infection rate in presence of this non-linearity. To our knowledge, no study has comprehensively evaluated the association of mobility with multiple phases of the pandemic growth pattern. This analysis becomes important as public health officials or policymakers can potentially use mobility as a predictor to detect impending local spikes of cases that are increasingly beginning to overwhelm clinical capacity.

This study proposes the association of mobility with the incidence growth rate of COVID− 19 by segmenting different infection growth rates into multiple phases. We used the formulations of different phases of Batista and Wu’s logistic growth model [25, 26] to extract the phases of the epidemic curve for various states in the United States. The United States presents a unique scenario since the peaks occurred across distinct epochs, within different geographic regions with independent administrative units. Thus, influenced by sets of multiple factors. We specifically study how mobility affected the case growth for various states following the lifting of initial stay-at-home orders.

## Methods

### Data collection and analysis

#### Infection data

The confirmed case data was retrieved from the data from The New York Times [27] and JHU CSSE COVID− 19 data [28]. This data provides county-level information on daily reported cases. We aggregated the data to weekly reports at the state level.

#### Mobility data

State-level mobility datasets and metrics were provided by Descartes Labs. Descartes index provides a normalized aggregated mobility measure obtained from anonymized mobile device locations. The mobility metric we use is the percentage change in mobility relative to pre-pandemic baseline behavior (02/17/2020 to 03/07/2020) [7].

#### Infection growth rate

We use the infection growth rate from the Classical Logistic Growth Model, originally developed by Haberman [29]. The logistic growth model is defined by the differential eq. 1.

1 $$ \frac{dC(t)}{dt}= rC(t)\left(1-\frac{C(t)}{K}\right) $$

C(t) is the cumulative number of confirmed cases at any given time t for an individual state, and $\frac{dC(t)}{dt}$ is the rate of change in the number of cases. The intrinsic growth rate per unit time *r* and the expected epidemic capacity *K* are estimated from the cases recorded over time using non-linear least square curve fitting methods, namely Levenberg-Marquardt, Trust Region Reflective Method, and Nelder-Mead methods that are available as part of the LMFIT Python package [30]. The peak of the curve (i.e., inflection point) is denoted by *t*_*p*_ is when $\frac{d^2C}{d{t}^2}$ and the number of cases *C*_*p*_ = *K*/2.

#### Phase-wise correlation

Our central idea is to separate the epidemic curve (which we also refer to as the peak) into multiple phases (or intervals) for the model, rather than fit the correlation between mobility and for the entire epidemic curve. We adopt a piece-wise correlation to study how the correlation varies across these distinct phases of the epidemic curve. We draw our inspiration from the general idea of piece-wise correlation and conditional correlation (also referred to as time-varying correlation, or dynamic correlation) that has been applied in several domains such as image processing [31], econometrics [32, 33], and bioinformatics [34] in situations where the distribution of relationship between variables is non-linear, and as a result, the degree of correlation, slope, and intercept vary across space or time.

**Fig. 1:**
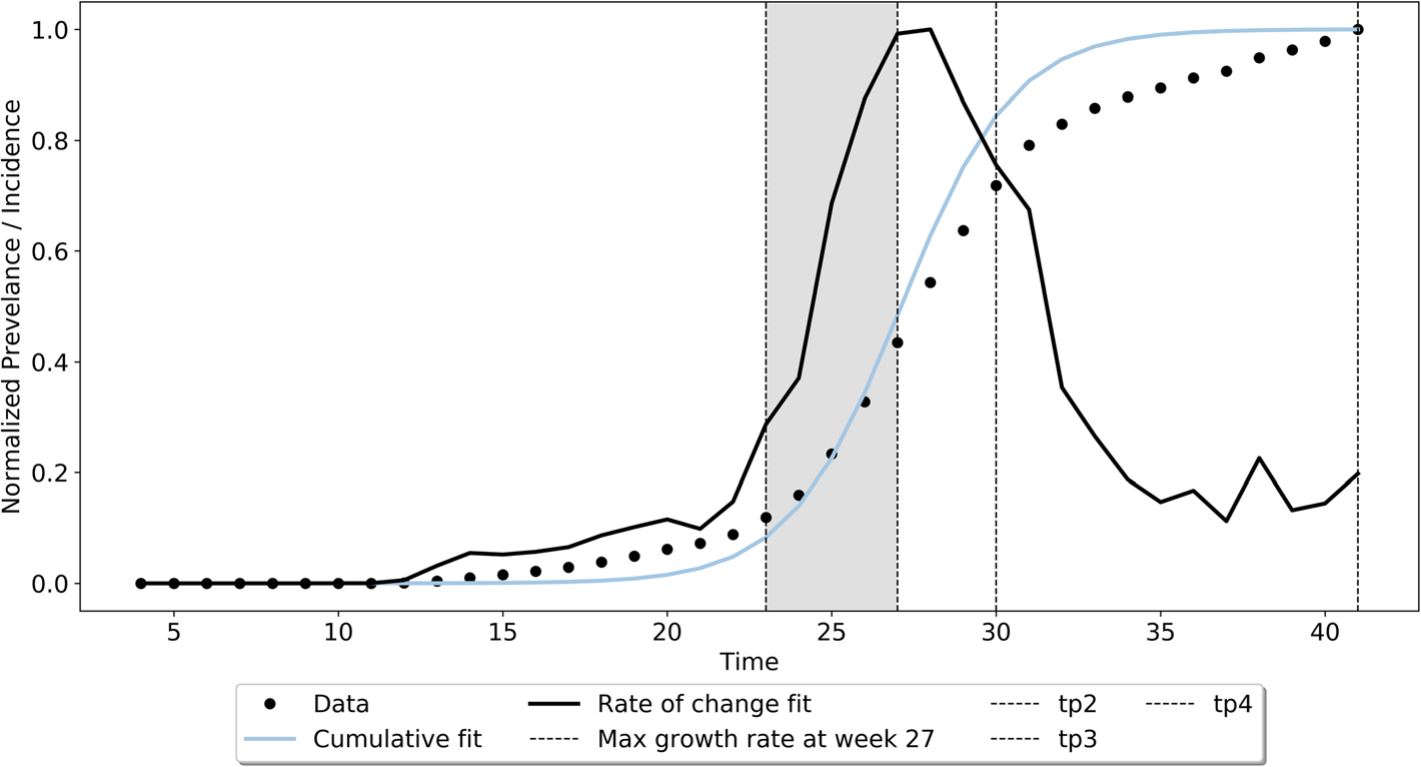
Various phases of the Logistic growth model for the State of Arizona

The separation of the epidemic curve into 5-phased intervals was done using the empirical approach adopted by Batista and Wu [25, 26] to separate the logistic growth curve generated. The phased intervals are as follows: (a) Phase-I is called the early growth phase (or ascending) where $t<{t}_p-\frac{2}{r}$ (b) Phase-II is the fast growth phase which falls between the end of the lag phase (or slow growth phase $t<{t}_p-\frac{2}{r}$) and the peak *t*_*p*_, i.e. ${t}_p-\frac{2}{r}<t<{t}_p$, (c) Phase-III is the fast growth to steady-state ${t}_p<t<{t}_p+\frac{2}{r}$, (d) Phase-IV – steady-state ${t}_p+\frac{2}{r}<t<2{t}_P$ and finally (e) Phase-V is the ending phase *t* > 2*t*_*p*_. These phases are illustrated for the logistic growth curve for the State of Arizona in Fig. 1.

The peak time *t*_*p*_ is at the 27th week, the slow growth phase is until in *t* = 22 weeks, and the fast growth phase, shown in grey shading is between 23rd to 27th week. While theoretically five phases have been defined, in a practical setting, Phase-V is only apparent in the post-hoc analysis of the epidemic. Given that many states have multiple peaks or constant changes in incidence growth rate, Phase-V tail ends into the Phase-I of the next peak and is considered the start of the second peak of the epidemic. Given that our focus is to understand if increased mobility affects growth in the number of cases, we limited our analysis for Phase− 1 to Phase-IV. Our sample includes all the 20 states that have completed a peak after the relaxation of initial stay-at-home orders (i.e. between July and September 2020). Before July, i.e. between March and June 2020, 14 states had a peak. Several other states continued to have peaks after September, but we limited our analysis to states that had a complete peak before September.

The cross-correlation between mobility and growth rate for various time lags for each phase *p* is obtained using Pearson’s correlation coefficient (provided in equation (eq. 2)) to compute the monotonic relationship between the two variables, incidence growth rate *I* and mobility index *M* for various phases (or intervals) of the logistic growth model.

2 $$ corr\left({I}_p,{M}_p\right)=\frac{\sum \limits_{i=0}^{n_p}\left({I}_{p,i}-\overline{I_{p,i}}\right)\left({M}_{p,i}-\overline{M_{p,i}}\right)}{\sqrt{\sum \limits_{i=0}^{n_p}{\left({I}_{p,i}-\overline{I_{p,i}}\right)}^2{\left({M}_{p,i}-\overline{M_{p,i}}\right)}^2}} $$

where *I*_*p*_ is the value of the incidence growth rate during phase *p*, and *M*_*p*_ is the lagged change in mobility during the phase *p*. *I*_*p*, *i*_ and *M*_*p*, *i*_ are the incidence growth rate and mobility rate at each sample point *i*, and *n*_*p*_ is the total number of weeks during the phase *p*.

## Results

### Mobility trends of various states

**Fig. 2:**
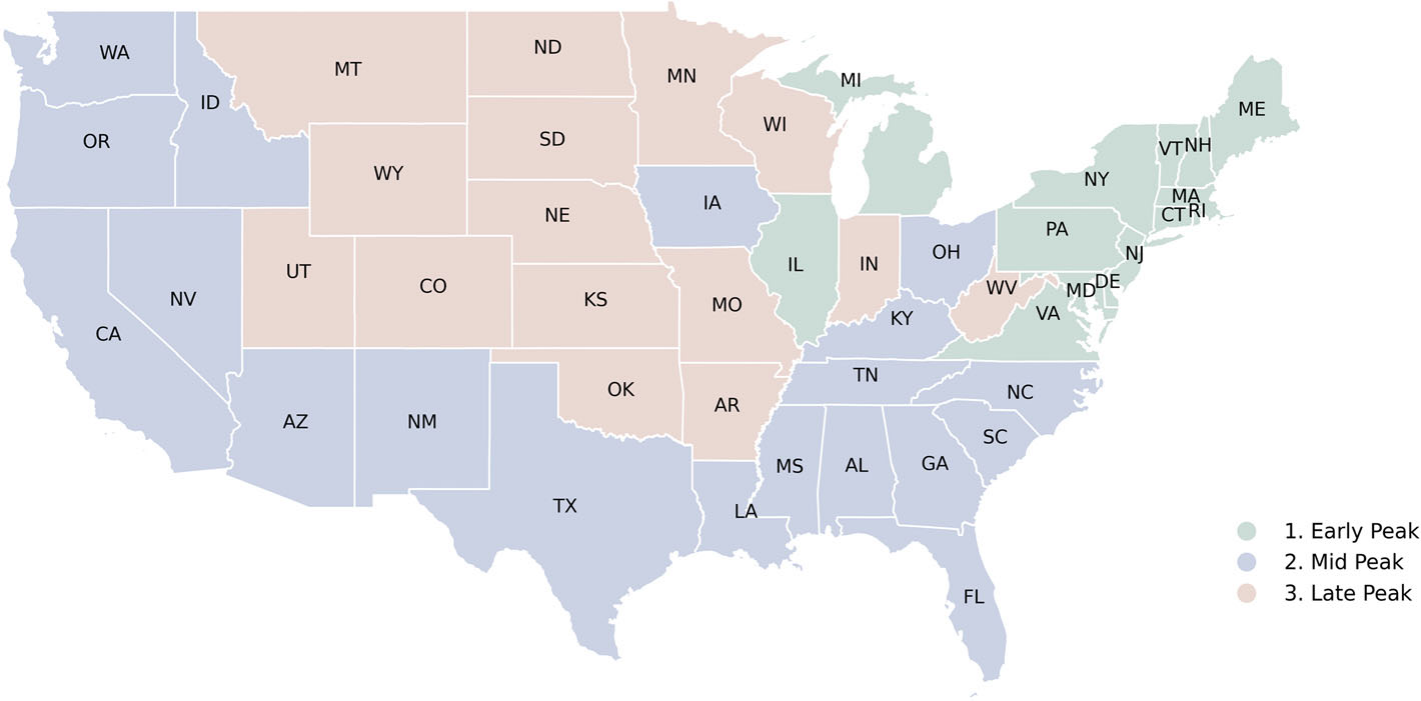
States classified according to whether they had an early peak (before July), mid peak (July to August), and late peak, (September onwards)

The states were separated into three categories (as shown in Fig. 2). The states that had an early surge between March and June (early peak), states that peaked in July and August (mid peak), and the states that peaked after August (late peak). It is worth noting that (based on data until November 30) the groups of states that have reached their peak in these epochs have been largely contiguous i.e., they clustered in space. The northeast peaked early; states like New York and New Jersey peaked in March and April. The rest of the coast peaked in summer, with states like Louisiana and Florida leading the outbreak front. The midlands peaked last in Autumn and Winter.

**Fig. 3:**
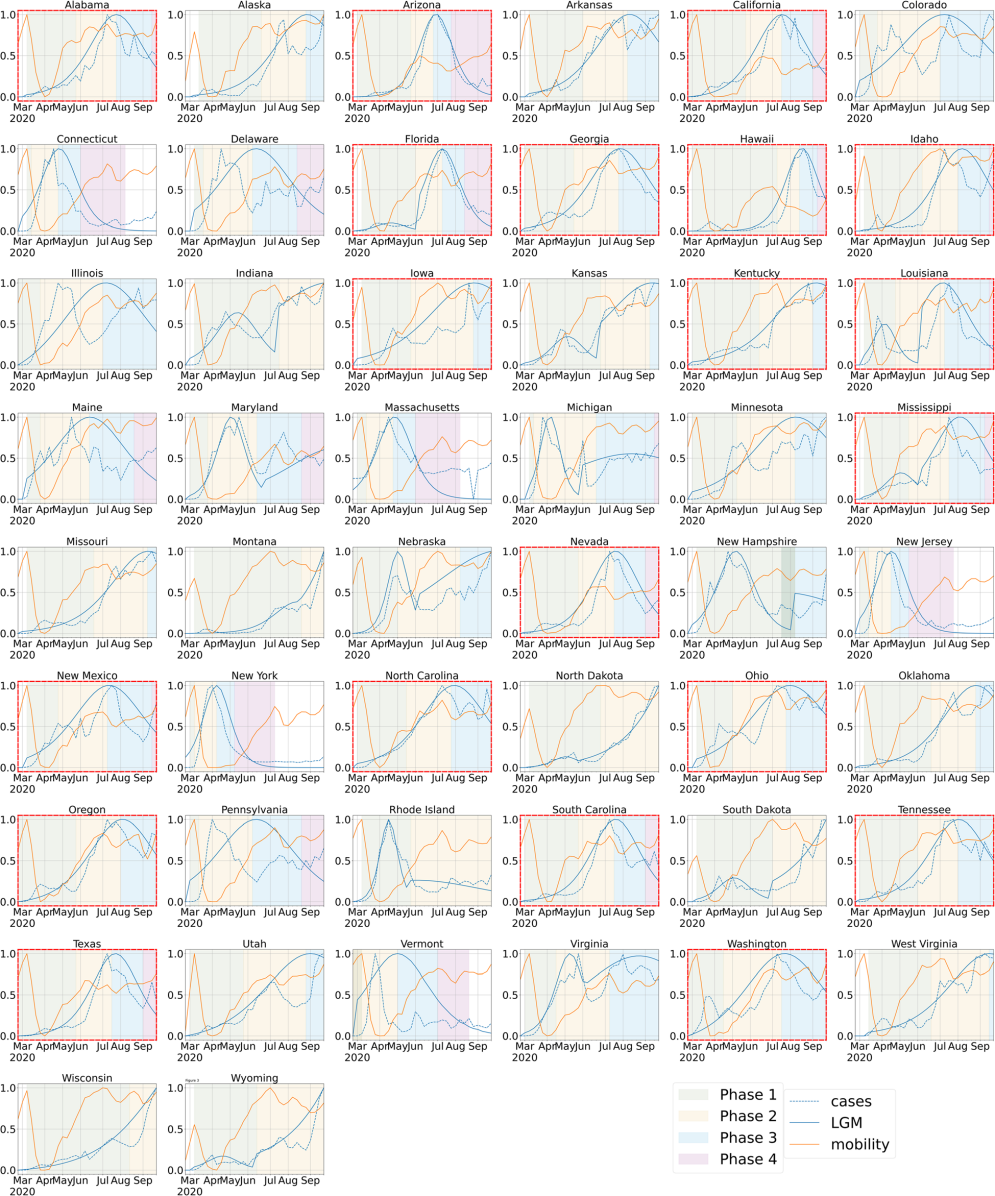
Mobility, Incidence, and Growth phases across states with the July–August peaking states highlighted

Figure 3 shows the mobility, reported cases, the case growth rate for all the 50 states in alphabetical order. We notice that there are multiple surges (which we refer to as peaks). As discussed previously, when there is a surge in a state, public health officials respond through non-pharmaceutical interventions to flatten the curve. Three significant points were used to model the peak in each state. The inflection point (the tip of the peak) is the time when the infection rate reaches the highest number. The point of transition to the fast-growth phase (i.e. Phase-II) is the point where the infection growth rate begins to transition from the slow growth phase (Phase-I) to the fast-growth phase. Finally, transition to Phase-III is where the infection growth rate transitions from the fastest deceleration phase to the slow deceleration phase. These phases are shown in Fig. 3. The states that had completed a peak (i.e. had at least all the 3 phases) between July and September were highlighted in a red dashed border. The background of each state plot is colored by the corresponding epidemic phase of the state. The three trend lines shown for each state are a.) the incidence of cases b.) an automated piece-wise logistic growth model fitted to the incidence of cases and c.) the mobility in the state as measured by Descartes Index. All the 3 trend lines have been applied min-max scaling to share common axes. The X-Axis stands for time, marked by months for major ticks and weeks for minor ticks. The Y-Axis stands for the scaled values of each series.

Of the 50 states in the United States, 14 states reported their peak number of cases early in the US epidemic during April and May, 20 states had their peak during July and August which are considered in the scope of our study and the other 16 states were still reporting an increasing number of cases in September. It should be noted that while the 20 states had their peak during July and August, their Phase-II may have started as soon as May, and their Phase-III may have ended as late as October. For example, the fast-growth rate phase for the state of New Mexico spanned from May to July. Similarly, the State of Iowa’s Phase-III extended until November.

### Relationship between mobility trends and infection growth-rate

The lifting of stay-at-home-order decisions varied across different states with no national mandate. States had variations in terms of prior infections, non-pharmaceutical interventions such as face masks, reopening guidelines, and population density. This combination of factors resulted in complex variations in mobility and infection growth rate across different states. Performing a linear continuation correlation across mobility and case growth rate did not yield any consistent correlations. This was also reported by Gatalo et al. [21].

We adopt a piece-wise association analysis approach to capture the non-linear nature of infection growth and understand if the correlations are associated with a particular phase. We also investigate the time lags between mobility and infection rates for various phases. The correlation was not consistently present across phases, and when correlation exists, it is not consistent within each phase. Table 1 shows the correlation between mobility and infection growth with a lag of 5 to 7 weeks. The correlation observations Phase-I, Phase-II, and Phase-II are not necessarily consistent. The average correlation is observed to be 0.15 ± 0.47 for the lag of 5 weeks between the change in mobility and the incidence rate of the cases. The similar number for Phases I, II, III, and IV are −0.49 ± 0.5, −0.17 ± 0.57, −0.32 ± 0.43, and − 0.33 ± 0.86, respectively. The associations are much stronger for Phase-II for states that peaked during the July and August of the US epidemic compared to the rest of the phases. Another interesting observation is that for phase-I, 13 out of 20 states have a strong negative correlation for a 6-week lag. This variation is due to a wide range of factors related to stay-at-home orders, prior infection seeding in communities, and increased testing rates. But the dominant factor for negative correlation is likely because the mobility numbers for 5 to 7-week lag, falls within March and April, where several states had mobility going down due to stay-at-home orders.

**Table 1:** Correlation analysis for all the states from March to September

State	Lag									
	1 Week	2 Weeks	3 Weeks	4 Weeks	5 Weeks	6 Weeks	7 Weeks	8 Weeks	9 Weeks	10 Weeks
Alabama	0	0	0	0.01	0.0	0.04	0.1	0.27	0.73	0.81
Alaska	0	0	0	0	0	0	0	0	0	0
Arizona	0.91	0.9	0.95	0.86	0.6	0.32	0.11	0.03	0	0
Arkansas	0	0	0	0	0	0	0.01	0.02	0.06	0.17
California	0.71	0.77	0.87	0.93	0.71	0.48	0.25	0.09	0.03	0
Colorado	0.02	0.06	0.15	0.4	0.79	0.71	0.3	0.08	0.01	0
Connecticut	0	0	0	0	0	0.07	0.37	0.88	0.68	0.43
Delaware	0.39	0.15	0.04	0.01	0	0	0	0	0	0
Florida	0.11	0.11	0.1	0.15	0.21	0.33	0.63	0.79	0.22	0.02
Georgia	0	0.01	0.01	0.02	0.04	0.1	0.24	0.52	0.96	0.53
Hawaii	0.51	0.72	0.99	0.74	0.52	0.37	0.29	0.29	0.37	0.58
Idaho	0	0	0	0	0	0	0	0	0.01	0.08
Illinois	0.1	0.24	0.48	0.93	0.61	0.26	0.07	0.01	0	0
Indiana	0.25	0.36	0.51	0.48	0.35	0.19	0.05	0.01	0	0
Iowa	0	0	0	0	0	0	0	0	0.01	0.03
Kansas	0.07	0.07	0.06	0.03	0.02	0.01	0	0	0	0
Kentucky	0	0	0	0	0	0	0	0.01	0.01	0.02
Louisiana	0.41	0.25	0.06	0.02	0	0.01	0.06	0.4	0.73	0.09
Maine	0.81	0.66	0.27	0.07	0.01	0	0	0	0	0
Maryland	0	0	0	0	0.07	0.72	0.23	0.01	0	0
Massachusetts	0	0	0	0	0.01	0.13	0.56	0.86	0.45	0.25
Michigan	0.08	0.38	0.7	0.04	0	0	0	0.01	0.23	0.73
Minnesota	0	0	0	0	0.01	0.02	0.04	0.12	0.29	0.6
Mississippi	0.01	0.02	0.02	0.01	0.01	0	0	0.01	0.03	0.21
Missouri	0.01	0.01	0.01	0.01	0.01	0.01	0.01	0.02	0.03	0.04
Montana	0	0	0	0	0	0	0	0	0	0
Nebraska	0.14	0.41	0.73	0.89	0.75	0.25	0.01	0	0	0
Nevada	0.16	0.19	0.26	0.4	0.62	0.95	0.63	0.28	0.08	0.01
New Hampshire	0	0	0	0	0	0.01	0.18	0.87	0.4	0.08
New Jersey	0	0	0	0.05	0.41	0.88	0.34	0.12	0.06	0.03
New Mexico	0.14	0.2	0.32	0.54	0.82	0.82	0.45	0.19	0.06	0.01
New York	0	0	0.06	0.47	0.77	0.28	0.1	0.05	0.04	0.03
North Carolina	0.02	0.02	0.04	0.07	0.13	0.24	0.44	0.76	0.78	0.36
North Dakota	0.01	0.01	0.01	0	0	0	0	0	0	0
Ohio	0	0	0	0	0.01	0.04	0.09	0.24	0.54	0.93
Oklahoma	0.01	0.01	0.01	0.01	0.01	0.01	0.01	0.01	0.02	0.04
Oregon	0	0	0	0.01	0.03	0.08	0.21	0.46	0.86	0.63
Pennsylvania	0.48	0.18	0.05	0.01	0	0	0	0	0	0
Rhode Island	0	0	0	0.01	0.43	0.42	0.08	0.09	0.29	0.76
South Carolina	0.02	0.02	0.03	0.06	0.12	0.22	0.44	0.8	0.65	0.22
South Dakota	0.08	0.06	0.04	0.01	0	0	0	0	0	0
Tennessee	0	0	0	0	0.01	0.02	0.04	0.12	0.31	0.71
Texas	0.09	0.11	0.13	0.18	0.26	0.4	0.62	0.94	0.66	0.28
Utah	0	0	0	0	0	0.01	0.01	0.02	0.05	0.08
Vermont	0	0	0	0	0	0	0.01	0.06	0.15	0.29
Virginia	0.99	0.59	0.3	0.16	0.13	0.3	0.87	0.03	0	0
Washington	0.01	0.02	0.07	0.2	0.43	0.77	0.78	0.37	0.11	0.02
West Virginia	0.01	0.01	0.01	0.01	0.01	0.01	0.01	0.01	0.02	0.02
Wisconsin	0	0	0	0	0	0	0	0	0	0
Wyoming	0.01	0	0	0	0	0	0	0	0	0

These results demonstrate that the piece-wise correlations capture the relationship between incidence growth rate and the change in mobility more accurately than performing linear and consistent correlation across multiple phases.

Table 2 shows the correlation between the case incidence growth rate and the change in mobility for Phase I of the pandemic during the study period. States that had a longer Phase-I and Phase-II like Idaho and Iowa, have a higher correlation compared to states like Arizona and Nevada whose epidemic growth phases are considerably quicker. Tables 3 and 4 show the correlations for all 50 states for various lag periods for Phases II and III.

**Table 2:** Correlation analysis for Phase I for all the states with multiple lags

State	Lag									
	1 week	2 weeks	3 weeks	4 weeks	5 weeks	6 weeks	7 weeks	8 weeks	9 weeks	10 weeks
Alabama	0.58	0.89	0.56	0.6	0.03	0	0	0.03	0.5	0.6
Alaska	0	0.04	0	0.36	0.67	0.66	0.37	0.35	0	0.06
Arizona	0.48	0.5	0	0.07	0.03	0.03	0.02	0.03	0	0.53
Arkansas	0.7	0.88	0.65	0.55	0.8	0.05	0.03	0.02	0.05	0.7
California	0	0.04	0.03	0.02	0	0	0	0.03	0	0.46
Colorado	0.6	0.55	0.53							
Connecticut										
Delaware	0.9	0.56								
Florida	0	0	0	0.23	0.78	0.09	0	0	0.02	0.6
Georgia	0.54	0.33	0.1	0.02	0.01	0.01	0.02	0.08	0.43	0.6
Hawaii	0.25	0.33	0.41	0.59	0.82	0.86	0.54	0.32	0.11	0.06
Idaho	0.16	0.35	0.68	0.82	0.52	0.2	0.07	0.05	0.06	0.11
Illinois	0.08	0.39	0.69	1						
Indiana	0.36	0.12	0.02	0.01	0.01	0.03	0.27	0.67	0.45	0.23
Iowa	0.82	0.33	0.27	0.13	0.04	0.02	0.03	0.06	0.17	0.59
Kansas	0.44	0.15	0.11	0.16	0.07	0.67	0.86	0.31	0.11	0.18
Kentucky	0.32	0.65	0.83	0.54	0.23	0.14	0.05	0.01	0.01	0.01
Louisiana	0	0.03	0.39	0.59	0.01	0.29	0.58	1		
Maine	0.03	0.59	0.51	1						
Maryland	0.07	0.39	0.63	1						
Massachusetts	0.7	1								
Michigan	1	1								
Minnesota	0.15	0.09	0.03	0.01	0.02	0.04	0.15	0.53	0.57	1
Mississippi	0.28	0.05	0	0	0.01	0.11	0.55	0.56	0.01	0.71
Missouri	0.14	0.41	0.74	0.78	0.61	0.27	0.2	0.07	0.02	0.01
Montana	0	0	0	0	0	0	0	0	0	0.01
Nebraska	0.22	0.05	0.01	0.01	0.02	0.15	0.6	0.63	1	
Nevada	0.21	0.08	0.05	0.03	0.02	0.02	0.02	0.03	0.1	0.5
New Hampshire	0	0	0	0	0	0.03	0.31	0.95	0.32	0.07
New Jersey	0.72	1								
New Mexico	0.03	0.01	0.02	0.05	0.18	0.71	0.45	1		
New York	1									
North Carolina	0.31	0.19	0.07	0.02	0.01	0.01	0.02	0.11	0.52	0.57
North Dakota	0.1	0.28	0.62	0.91	0.99	0.54	0.26	0.07	0.25	0.7
Ohio	0.16	0.03	0.01	0.02	0.03	0.12	0.51	0.62	1	
Oklahoma	0.08	0.18	0.4	0.83	1	0.52	0.38	0.12	0.03	0.01
Oregon	0.26	0.09	0.07	0.03	0.02	0.02	0.02	0.04	0.14	0.63
Pennsylvania	0.58	1								
Rhode Island	0.01	0	0.03	0.37	0.54	0.03	0	0.03	0.29	0.94
South Carolina	0.97	0.68	0.43	0.14	0.03	0.01	0.01	0.03	0.14	0.62
South Dakota	0.23	0.09	0.01	0.01	0.02	0.07	0.88	0.47	0.09	0
Tennessee	0.83	0.97	0.58	0.34	0.1	0.02	0.01	0.01	0.02	0.09
Texas	0.7	0.37	0.29	0.12	0.05	0.02	0.01	0.02	0.09	0.46
Utah	0.73	0.39	0.31	0.18	0.11	0.12	0.19	0.32	0.64	0.85
Vermont										
Virginia	0.02	0.01	0.02	0.08	0.46	0.61	1			
Washington	0.01	0.01	0.03	0.11	0.52	0.69	1			
West Virginia	0.01	0.18	0.53	0.99	0.71	0.35	0.19	0.06	0.01	0.01
Wisconsin	0	0	0	0	0	0	0.02	0.04	0.11	0.27
Wyoming	0.13	0.18	0.15	0.58	0.99	0.25	0.04	0.05	0.55	0.92

**Table 3:** Correlation analysis for Phase II for all the states with multiple lags

State	Lag									
	1 week	2 weeks	3 weeks	4 weeks	5 weeks	6 weeks	7 weeks	8 weeks	9 weeks	10 weeks
Alabama	0.11	0.01	0.01	0.01	0	0	0	0	0	0.03
Alaska	0.4	0.94	0.62	0.58	0.31	0.04	0	0	0	0
Arizona	0.72	0.1	0.01	0.02	0.02	0.06	0.01	0.04	0.82	0.09
Arkansas	0.68	0.42	0.04	0.01	0	0	0	0	0	0
California	0.12	0	0	0	0	0	0.01	0.03	0.23	0.13
Colorado	0	0	0.1	0.99	0.46	0.2	0.1	0.03	0.02	0.01
Connecticut	0.06	0	0.01	0.05	0.22	0.7	0.48	1	−1	−1
Delaware	0.05	0.5	0.06	0.01	0.01	0.01	0	0.01	0.02	0.08
Florida	0.04	0.09	0	0.01	0.02	0	0	0	0.02	0.19
Georgia	0	0	0	0	0	0	0	0	0.02	0.8
Hawaii	0.02	0.01	0.01	0.12	0.73	0.01	0.01	0	0.01	0
Idaho	0.6	0.26	0	0	0	0	0	0	0	0
Illinois	0	0	0.21	0.67	0.17	0.06	0.03	0.01	0.01	0.01
Indiana	0.63	0.33	0.12	0.16	0.13	0.21	0.43	0.35	0.08	0
Iowa	0.04	0.01	0	0	0	0	0	0	0	0
Kansas	0.04	0.06	0.02	0.02	0.92	0.53	0.06	0	0	0
Kentucky	0.08	0.15	0.94	0.37	0.05	0.01	0	0	0	0
Louisiana	0.01	0.03	0.01	0.01	0	0	0.05	0.48	0.66	0.17
Maine	0	0	0.62	0.4	0.09	0.04	0.01	0.01	0.01	0.03
Maryland	0.09	0.01	0.02	0.04	0.16	0.74	0.42	0.05	0.01	0
Massachusetts	0.06	0.01	0.01	0.03	0.17	0.59	0.54	1	−1	−1
Michigan	0.04	0.23	0.98	0.07	0	0	0.01	0.19	0.78	0.04
Minnesota	0	0	0	0	0	0	0	0	0.21	0.69
Mississippi	0.01	0.01	0.01	0.01	0	0	0	0	0.01	0.63
Missouri	0.03	0.05	0.11	0.71	0.37	0.05	0.01	0	0	0
Montana	0.12	0.08	0.02	0.55	0.69	0.6	0.35	0.18	0.08	0.79
Nebraska	0.75	0.83	0.88	0.89	0.92	0.59	0.18	0	0	0
Nevada	0.08	0	0	0	0	0	0	0	0.05	0.44
New Hampshire	−1	−1	−1	−1	−1	−1	−1	−1	−1	−1
New Jersey	0.05	0	0.03	0.18	0.61	0.53	1	−1	−1	− 1
New Mexico	0	0	0	0	0	0.03	0.71	0.11	0.02	0.01
New York	0.01	0.05	0.23	0.74	0.43	1	−1	−1	−1	−1
North Carolina	0	0	0	0	0	0	0	0	0.07	0.78
North Dakota	0.01	0.01	0.02	0.15	0.96	0.59	0.27	0.09	0.02	0
Ohio	0	0	0	0	0	0	0	0.24	0.59	0.21
Oklahoma	0.04	0.01	0	0.74	0.89	0.08	0.01	0	0	0
Oregon	0.02	0	0	0	0	0	0	0	0	0.19
Pennsylvania	0.7	0.22	0.06	0.02	0.01	0.01	0	0.01	0.02	0.07
Rhode Island	0.13	0.05	0.02	0	0	0	0	0	0	0
South Carolina	0.03	0.02	0.04	0.01	0.01	0	0	0	0	0.2
South Dakota	0	0	0	0.11	0.73	0.2	0.01	0.01	0	0
Tennessee	0.56	0.08	0	0	0	0	0	0	0	0
Texas	0.46	0.11	0.05	0	0	0	0	0	0	0.21
Utah	0.01	0	0	0	0	0	0	0	0	0
Vermont	0	0	0	0.01	0.04	0.18	0.57	0.44	1	−1
Virginia	0.71	0.75	0.7	0.39	0.1	0.05	0.3	0.53	0	0
Washington	0	0	0	0	0	0.68	0.23	0.04	0.02	0.01
West Virginia	0.06	0.03	0.05	0.67	0.78	0.18	0.03	0	0	0
Wisconsin	0.24	0.59	0.82	0.21	0.31	0.5	0.09	0.02	0.15	0.84
Wyoming	0	0.03	0.29	0.65	0.15	0.03	0	0	0	0

**Table 4:** Correlation analysis for Phase II for all the states with multiple lags

State	Lag									
	1 week	2 weeks	3 weeks	4 weeks	5 weeks	6 weeks	7 weeks	8 weeks	9 weeks	10 weeks
Alabama	0.11	0.01	0.01	0.01	0	0	0	0	0	0.03
Alaska	0.4	0.94	0.62	0.58	0.31	0.04	0	0	0	0
Arizona	0.72	0.1	0.01	0.02	0.02	0.06	0.01	0.04	0.82	0.09
Arkansas	0.68	0.42	0.04	0.01	0	0	0	0	0	0
California	0.12	0	0	0	0	0	0.01	0.03	0.23	0.13
Colorado	0	0	0.1	0.99	0.46	0.2	0.1	0.03	0.02	0.01
Connecticut	0.06	0	0.01	0.05	0.22	0.7	0.48	1	−1	−1
Delaware	0.05	0.5	0.06	0.01	0.01	0.01	0	0.01	0.02	0.08
Florida	0.04	0.09	0	0.01	0.02	0	0	0	0.02	0.19
Georgia	0	0	0	0	0	0	0	0	0.02	0.8
Hawaii	0.02	0.01	0.01	0.12	0.73	0.01	0.01	0	0.01	0
Idaho	0.6	0.26	0	0	0	0	0	0	0	0
Illinois	0	0	0.21	0.67	0.17	0.06	0.03	0.01	0.01	0.01
Indiana	0.63	0.33	0.12	0.16	0.13	0.21	0.43	0.35	0.08	0
Iowa	0.04	0.01	0	0	0	0	0	0	0	0
Kansas	0.04	0.06	0.02	0.02	0.92	0.53	0.06	0	0	0
Kentucky	0.08	0.15	0.94	0.37	0.05	0.01	0	0	0	0
Louisiana	0.01	0.03	0.01	0.01	0	0	0.05	0.48	0.66	0.17
Maine	0	0	0.62	0.4	0.09	0.04	0.01	0.01	0.01	0.03
Maryland	0.09	0.01	0.02	0.04	0.16	0.74	0.42	0.05	0.01	0
Massachusetts	0.06	0.01	0.01	0.03	0.17	0.59	0.54	1	−1	−1
Michigan	0.04	0.23	0.98	0.07	0	0	0.01	0.19	0.78	0.04
Minnesota	0	0	0	0	0	0	0	0	0.21	0.69
Mississippi	0.01	0.01	0.01	0.01	0	0	0	0	0.01	0.63
Missouri	0.03	0.05	0.11	0.71	0.37	0.05	0.01	0	0	0
Montana	0.12	0.08	0.02	0.55	0.69	0.6	0.35	0.18	0.08	0.79
Nebraska	0.75	0.83	0.88	0.89	0.92	0.59	0.18	0	0	0
Nevada	0.08	0	0	0	0	0	0	0	0.05	0.44
New Hampshire	−1	−1	−1	−1	-1	-1	-1	-1	-1	-1
New Jersey	0.05	0	0.03	0.18	0.61	0.53	1	-1	-1	-1
New Mexico	0	0	0	0	0	0.03	0.71	0.11	0.02	0.01
New York	0.01	0.05	0.23	0.74	0.43	1	-1	-1	-1	-1
North Carolina	0	0	0	0	0	0	0	0	0.07	0.78
North Dakota	0.01	0.01	0.02	0.15	0.96	0.59	0.27	0.09	0.02	0
Ohio	0	0	0	0	0	0	0	0.24	0.59	0.21
Oklahoma	0.04	0.01	0	0.74	0.89	0.08	0.01	0	0	0
Oregon	0.02	0	0	0	0	0	0	0	0	0.19
Pennsylvania	0.7	0.22	0.06	0.02	0.01	0.01	0	0.01	0.02	0.07
Rhode Island	0.13	0.05	0.02	0	0	0	0	0	0	0
South Carolina	0.03	0.02	0.04	0.01	0.01	0	0	0	0	0.2
South Dakota	0	0	0	0.11	0.73	0.2	0.01	0.01	0	0
Tennessee	0.56	0.08	0	0	0	0	0	0	0	0
Texas	0.46	0.11	0.05	0	0	0	0	0	0	0.21
Utah	0.01	0	0	0	0	0	0	0	0	0
Vermont	0	0	0	0.01	0.04	0.18	0.57	0.44	1	-1
Virginia	0.71	0.75	0.7	0.39	0.1	0.05	0.3	0.53	0	0
Washington	0	0	0	0	0	0.68	0.23	0.04	0.02	0.01
West Virginia	0.06	0.03	0.05	0.67	0.78	0.18	0.03	0	0	0
Wisconsin	0.24	0.59	0.82	0.21	0.31	0.5	0.09	0.02	0.15	0.84
Wyoming	0	0.03	0.29	0.65	0.15	0.03	0	0	0	0

## Discussion

Monitoring mobility trends could potentially inform mitigation measures towards slowing the spread of COVID-19. It can help predict the fast growth phase with exponential growth. The disparities in mobility and case incidence rate across the country, during fall and winter, indicate high variability in mitigation measures and pandemic behavior in various states across the United States. Given the non-linear nature of both mobility and case growth trends, we adopted a piece-wise approach to analyze the association between mobility and case growth rate.

Both mobility and the dynamic of epidemic spread vary quite widely in many aspects. First, the growth and lag dynamics are different across different scales, i.e. days, weeks, or months. Our choice of choosing weekly case numbers was motivated by the need to account for testing delays and the need to have an adequate sample size. We need to further investigate how the growth patterns vary across different temporal granularities. Second, we can observe that the growth curve patterns also differ across states. This difference is due to a combination of factors ranging from population density, differences in the actions of state and local authorities that introduce restrictions, and differences in how people adhere to social distancing restrictions in case of prolonged stay-at-home orders. Finally, mobility dynamics vary widely across different states. We observe that in most cases. The data until November shows that states with low mobility had lower per-capita cases, whereas most states had case increase in November irrespective of stay-at-home orders or mobility. These combinations of factors affect the relationship between mobility and infection growth rate.

This study uses an interval (or piece-wise) approach to address the non-linear trend arising from non-pharmaceutical interventions and pandemic behavior to flatten the curve. We used a Logistic Growth Model to separate the infection growth curve into multiple phases and apply correlation to individual phases. Most communities are experiencing multiple COVID-19 infection waves, as infection rates are modulated by lockdowns and other forms of non-pharmaceutical interventions followed by periods of relaxation.

**Fig. 4:**
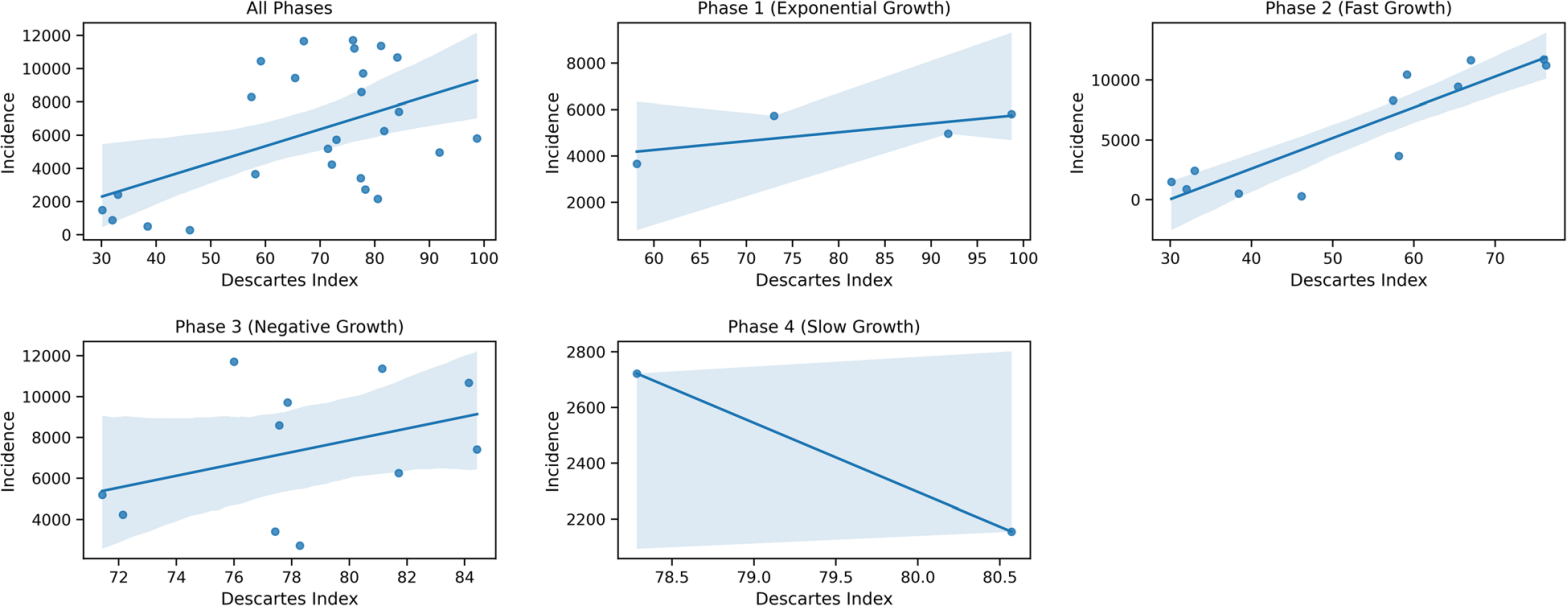
Correlation between incidence and mobility for Louisiana for various growth phases

We found that mobility has a strong correlation with and fast-growth phase with a lag of 5 to 7 weeks, but only in states with an early second peak. We found that the second peak characteristics differed in relation to the first peak and were consistently more protracted in their response to the mobility signal. When we examined the correlation between mobility and the number of cases for March to September, we observed that the correlations were not consistent (Fig. 4). In this representative figure, we show the relationship between the mobility index and the incidence growth values for the state of Louisiana with a lag of 6 weeks for the pandemic and each of the phases described in Fig. 1.

The lack of consistency between mobility and the number of cases for March to September could partly be due to variations in how states have relaxed social distancing guidelines over time. During the initial stay-at-home-order period, all the states underwent a sudden drop in mobility. This decrease in mobility correlated well with a drop in the number of cases for most states, after a lag period. However, the uptick in mobility in various states did not follow a consistent pattern after reopening. For instance, mobility in California stayed consistently below 60% until September. In contrast, states like Florida reopened quickly; with the mobility returning to as much as 80%. We did not observe a consistent positive correlation across multiple states during the relaxing of stay-at-home orders.

Prior literature explored a linear association between mobility and the number of cases. In this work, we argue that the distribution is not a simple linear trend, and instead we adopt a logistic growth model that is more faithful to the characteristics of the data and show the differential contribution of different phases which vary significantly amongst themselves, but also show consistent patterns that can be exploited in predicting subsequent spikes. In other words, a spike in mobility 5 to 7 weeks ahead may be an indicator of a state experiencing a peak. This advanced warning could potentially help states in advanced preparation for when hospitals may be overwhelmed.

As we see in this instance and most states with a co-temporal second peak, the correlation is much stronger in Phase-II compared to the other phases. The association between mobility and the number of cases is weaker in other phases. While the mobility decreases slightly after the number of cases increases, it is likely that the change in public behavior due to increases in social distancing, masks, testing, and other precautionary measures lead to a rapid decrease in the number of cases. More research is needed to confirm the influence of these factors, and to understand the reduction in the number of cases while the mobility stayed the same during the remaining phases of the pandemic in some instances.

Mobility is a useful indicator and publicly available mobility datasets from Safegraph and Descartes Labs can be leveraged in the early part of the pandemic to monitor population behavior in response to public health directives. However, their value wears off rapidly. We attempted a finer-grained analysis to examine whether the correlation is maintained at specific phases. While some interesting insights were gained, the analysis was not a practical tool that can generally predict the number of cases beyond the first peak due to a more noisy set of factors complicating the analytic space. The approach however may be useful in countries where a more strict and consistent set of mitigation directives have been applied, thus presenting a simpler analytic space.

## Limitations

This study presents a novel way to examine the association between mobility and infection rates for various states in the United States. There are several areas where this study can be potentially improved. First, this study focuses on the association between the change in mobility and its effect on the increases in the number of cases. This study does not take into effect, the many other factors like usage of masks, social distancing, the effect of regulations, and the varying compliance from the public that could have contributed to the number of cases. Without detailed information for each of these variables, it would not be possible to model for the causal effect of these factors in the incidence growth rate. Second, the associations are computed at a weekly granularity to overcome the non-uniform case reporting issues where a higher number of cases are reported over Mondays and Tuesdays while the number of cases reported over the weekend is lower. This leads to a sample size of 37 weeks during the study period, but as more data is collected, future studies can look at longer periods and larger sample sizes to validate these results. Third, the case data might be prone to reported errors due to both reporting issues as well as the outliers in testing when states update their case numbers post-hoc. We partly handle these issues by computing the incidence growth rate fitted to the Logistic Growth Model which smoothens the data, rather than the number of cases directly. Finally, the mobility data considers the distance traveled by individuals but does not capture the number of individuals making the trip. Incorporating the number of trips or individuals might help enhance the relationship between the number of cases and the mobility of individuals.

## Conclusion

We analyzed the trends in reported COVID-19 cases and mobility for various states in the United States. We noticed that the prior literature explored the correlation between a power-law distribution in the case distribution using simple linear modeling and we instead modeled using a Logistic Growth Model, more faithful to the distribution of the case data. This allowed us to perform multiple piece (phase)-wise linear correlations. We however found the associations to be quite asymmetrical across the phases.

Despite attempting to fit both linear and piece-wise correlations into the second wave, we were unable to find consistent patterns that would allow us to predict the rise in the number of cases. Although we note several insights in the distribution of the case data and its associations with mobility, we conclude that it is not productive to associate mobility with cases beyond the first peak. This is consistent with the findings of Gatalo et.al [21].

## Data Availability

The analysis code for this paper is available on GitHub at https://github.com/raviteja-bhupatiraju/Covid19-Phasewise-Mobility-Association
