# Correction to: Exploring the relationship between mobility and COVID− 19 infection rates for the second peak in the United States using phase-wise association

**DOI:** 10.1186/s12889-021-11904-4

**Published:** 2021-10-26

**Authors:** Raju Gottumukkala, Satya Katragadda, Ravi Teja Bhupatiraju, Azmyin Md. Kamal, Vijay Raghavan, Henry Chu, Ramesh Kolluru, Ziad Ashkar

**Affiliations:** 1 Informatics Research Institute, University of Louisiana at Lafayette, Lafayette, USA; 2 Mechanical Engineering Department, University of Louisiana at Lafayette, Lafayette, USA

**Correction to: BMC Public Health. 21, 1669 (2021)**

**http://orcid.org/10.1186/s12889-021-11657-0**

It was highlighted that in the original article [1] the two given names of author Azmyin Md. Kamal were erroneously interchanged. The original article has been updated.
